# Rapid new particle formation driven by methanesulfonic acid and amines

**DOI:** 10.1039/d5ea00081e

**Published:** 2026-07-06

**Authors:** Hannah Klebach, Lucía Caudillo-Plath, Martin Heinritzi, Douglas M. Russell, Birte Rörup, Rima Baalbaki, Jiali Shen, Eva Sommer, Andreas Kürten, Dina Alfaouri, Joao Almeida, António Amorim, Lisa J. Beck, Hannah Beckmann, Moritz Berntheusel, Theodoros Christoudias, Lubna Dada, Jenna DeVivo, Neil Donahue, Jonathan Duplissy, Imad El Haddad, Armin Hansel, Hartwig Harder, Xu-Cheng He, Markku Kulmala, Felix Kunkler, Katrianne Lehtipalo, Lu Liu, Bernhard Mentler, Tuukka Petäjä, Pedro Rato, Sarah Richter, Siegfried Schobesberger, Wiebke Scholz, Mario Simon, Roseline C. Thakur, António Tomé, Yandong Tong, Jens Top, Rainer Volkamer, Paul M. Winkler, Boxing Yang, Marcel Zauner-Wieczorek, Jiangyi Zhang, Jasper Kirkby, Joachim Curtius

**Affiliations:** a Institute for Atmospheric and Environmental Sciences, Goethe University Frankfurt Frankfurt am Main Germany klebach@iau.uni-frankfurt.de curtius@iau.uni-frankfurt.de; b Institute for Atmospheric and Earth System Research/Physics, Faculty of Science, University of Helsinki Helsinki Finland; c Climate and Atmosphere Research Centre (CARE-C), The Cyprus Institute Nicosia Cyprus; d Helsinki Institute of Physics, University of Helsinki Helsinki Finland; e CERN, The European Organization for Nuclear Research Geneva Switzerland; f Faculty of Physics, University of Vienna Wien Austria; g Faculdade de Ciências da Universidade de Lisboa Lisboa Portugal; h Laboratório de Instrumentação e Física Experimental de Partículas Lisboa Portugal; i Institute for Ion Physics and Applied Physics, University of Innsbruck Innsbruck Austria; j Laboratory of Atmospheric Chemistry, Paul Scherrer Institute Villigen Switzerland; k Center for Atmospheric Particle Studies, Carnegie Mellon University Pittsburgh PA USA; l Atmospheric Chemistry Department, Max Planck Institute for Chemistry Mainz Germany; m Finnish Meteorological Institute Helsinki Finland; n Department of Technical Physics, University of Eastern Finland Kuopio Finland; o Instituto Dom Luiz (IDL), Universidade da Beira Interior Covilhã Portugal; p Department of Chemistry, University of Colorado Boulder Boulder CO USA

## Abstract

The formation and radiative properties of clouds in the marine boundary layer are highly sensitive to the number of cloud condensation nuclei (CCN), which largely originate from new particle formation. At present, most climate models only consider new particle formation from sulfuric acid (SA), in pristine marine regions produced *via* oxidation of dimethyl sulfide (DMS) emitted by phytoplankton. However, DMS oxidation also yields methanesulfonic acid (MSA) – often in higher amounts than SA under cool conditions (<10 °C) – yet MSA's role in NPF remains elusive. Here, we present results from the CERN CLOUD chamber at temperatures of −10 °C and +5 °C, demonstrating NPF from MSA and amines (dimethylamine, DMA, and trimethylamine, TMA). We isolated effects of MSA from SA by generating MSA from an evaporator at concentrations between 10^5^ and 10^8^ cm^−3^. We find MSA and DMA form particles at +5 °C but nucleation rates (*J*_1.7_) are slow, reaching about 1 cm^−3^ s^−1^ at MSA concentrations of 5 × 10^7^ cm^−3^. However, in the presence of low concentrations of SA (below 10^6^ cm^−3^) and 2–15 pptv DMA, MSA at few 10^7^ cm^−3^ strongly enhances SA-DMA nucleation, reaching up to 80 cm^−3^ s^−1^. Our measurements confirm MSA together with SA and DMA molecules in initial molecular clusters during nucleation. We find TMA less effective than DMA for new particle formation, likely resulting from steric hindrance of the additional methyl group. Our findings show that MSA can boost NPF rates by 1–2 orders of magnitude in pristine marine environments and should be incorporated in climate models.

## Abstract 2

CERN CLOUD chamber measurements investigate the synergistic new particle formation rates and mechanisms involving methanesulfonic acid, sulfuric acid and two different amines under marine boundary layer conditions.

## Graphical abstract

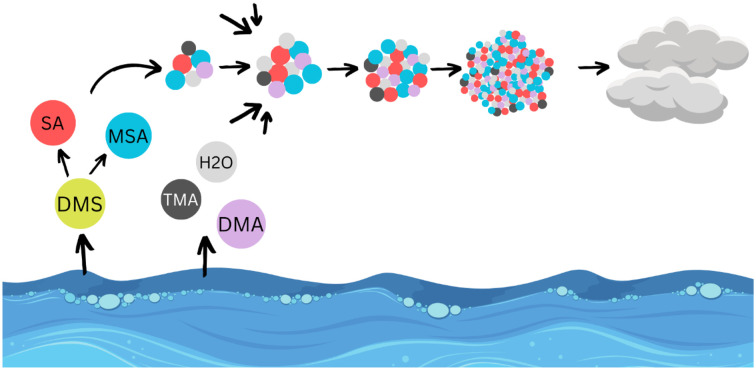

**Environmental significance**

Atmospheric new particle formation is responsible for more than half of global cloud condensation nuclei. Several precursor vapours have been identified beyond sulfuric acid, yet significant uncertainties remain before achieving a comprehensive understanding of aerosol formation, which is crucial for climate models. Here we report the nucleation potential of methanesulfonic acid in combination with two different amines at various atmospheric concentrations, and its synergy with sulfuric acid in multi-acid nucleation. Our findings indicate that MSA can be an important driver of new particle formation in cool, remote marine areas and in the pristine preindustrial atmosphere.

## 1 Introduction

Aerosols strongly influence Earth's radiation budget, both directly, by affecting incoming solar radiation, and indirectly, by influencing cloud properties such as albedo and lifetime. Our limited understanding of the aerosol formation processes and their interactions with clouds cause large uncertainties in current climate models.^1^ Hence, the fate of aerosols has been an important focus in atmospheric science for decades. Aerosols arise either by direct emission or by the condensation of vapours of extremely low volatility—known as ‘new particle formation’ (NPF). Once these particles grow to sufficient sizes (>50 nm) they can act as cloud condensation nuclei (CCN). Model studies show that around half of the global CCN originate from NPF.^2,3^ Due to the significant impact of airborne particles, their formation has been studied extensively and different gases have been identified as efficient nucleators depending on the atmospheric conditions. One of the most important species for nucleation is sulfuric acid (SA; H_2_SO_4_). Its high hydrogen bonding strength and relative abundance from both natural and anthropogenic sources with peak values of several 10^7^ cm^−3^ make it an important source of aerosols, as shown by field and chamber measurements.^4,5^ SA is thought to be the dominant nucleation precursor in many regions in the free troposphere as well as the boundary layer.^6,7^ However, since sulfuric acid and water alone cannot explain NPF observed in the boundary layer,^5^ additional stabilisers are required. This can, for example, arise from charge-stabilisation by ions from galactic cosmic rays (GCR).^5^ Another mechanism is acid–base stabilisation by co-condensation of ammonia or amines.^5,8,9^

Another atmospheric acid, found especially in marine environments, is methanesulfonic acid (MSA, CH_4_SO_3_). MSA and SA are two of the main oxidation products of dimethylsulfide (DMS, (CH_3_)_2_S), the dominant natural sulfur source to the atmosphere with an approximate flux of 18 to 24 Tg S year^−1^.^10^ The chemical structures of both acids differ slightly since one acidic proton is exchanged for a methyl group in MSA. This leads to a decreased H-bonding capacity and lower gas phase-acidity of MSA.^11^ The yield of MSA relative to SA increases at low temperatures, which makes MSA the major oxidation product from DMS at high latitudes or altitudes.^12^ MSA concentrations in marine environments can be comparable or higher than SA concentrations, with typical values of atmospheric observations ranging from 10^5^ cm^−3^ to 10^7^ cm^−3^.^13,14^ Even higher values were published by Yan *et al.*^15^ with mixing ratios of 3.3 pptv (corresponding to 8.8 × 10^7^ cm^−3^ at 0 °C ambient temperature and 1013 hPa pressure) in the Southern Ocean; maximum values reported were up to a factor of 7 higher. MSA concentrations above 1 × 10^7^ cm^−3^ were also found in the free troposphere and are interpreted as re-evaporation of MSA from aerosols in drier or warmer conditions.^16^ Mauldin *et al.*^17^ reported high nighttime concentrations of MSA of up to 2 × 10^7^ cm^−3^ in the remote Pacific.

MSA is known to drive particle growth^13,18^ and its ability to participate in new particle formation has been widely studied.^19–22^ However, experimental studies on the nucleation mechanisms and measurements of nucleation rates are very sparse. The first molecular study of MSA-NH_3_ nucleation and growth under atmospheric conditions was conducted at the CLOUD chamber by Baalbaki *et al.*^22^. The findings demonstrate that MSA was capable of nucleating with NH_3_ alone at −10 °C and −30 °C. Furthermore it was observed that MSA enhances the nucleation rate of SA-NH_3_ at these temperatures. At +10 °C no enhancement of the particle formation rate was observed and no pure MSA-NH_3_ clusters were detected. However, at +10 °C and below, MSA drives rapid growth of particles in the size range of 1.8 nm and above, reaching the kinetic limit, and was directly detected in particles as small as 8 nm.

Beyond ammonia, amines constitute a major group of bases contributing to atmospheric chemistry. Among the most abundant amines are methylamine, dimethylamine (DMA, (CH_3_)_2_NH) and trimethylamine (TMA, (CH_3_)_3_N).^23–25^ Amines can stabilise acidic clusters more strongly than ammonia due to their higher basicity. Studies show that DMA is nucleating with SA at the kinetic limit.^26^ Hence high nucleation rates above 1000 cm^−3^ s^−1^ can be reached even at amine mixing ratios below 10 pptv.^8,26^ The main anthropogenic sources of amines are animal husbandry (146 Gg N per year from methylamines) and biomass burning (60 Gg N per year from methylamines).^23^ Chen *et al.*^27^ and Gao *et al.*^28^ also reported amine emissions from the Chinese Sea, Dall’Osto *et al.*^29^ found alkylamines released from melted sea ice in the Antarctic, and van Pinxteren *et al.*^30^ measured up to 10 pptv DMA originating from the tropical Atlantic, indicating the importance of marine amine sources.

Since SA-amines have higher nucleation rates than SA-NH_3_, a similar behaviour could be expected for MSA. NPF studies using flow tubes have revealed particle formation from MSA and DMA, and reported a strong dependence on the relative humidity.^21,31,32^ However, the short residence time and high loss rates in flow tubes require vapour concentrations well above ambient values, which limit the application of these results in climate models. Modelling studies^33–35^ predict weak nucleation from MSA and DMA and even lower nucleation rates for TMA. In contrast, high particle formation rates are predicted for the mixed system of sulfuric and methanesulfonic acid together with amines. DMA and TMA are both expected to be more effective than ammonia, even at 1000-fold lower concentrations. In the gas phase TMA is more basic than DMA due to the presence of an additional methyl group. The nitrogen atom in TMA experiences more electron donation *via* the inductive effect and is more susceptible to accetping a proton. However, DMA is expected to be more effective for nucleation, as TMA lacks a polar N–H bond and exhibits increased steric hindrance due to the additional methyl group. Recent flow tube studies by Johnson and Jen^21^ confirm these model calculations. They found that MSA is interacting directly with DMA and can enhance the cluster formation when added to the SA-DMA system. In the case of MSA-SA-TMA, MSA was shown to suppress nucleation in high concentrations, likely due to the trapping of TMA in small clusters with MSA and thereby preventing further reactions with SA or MSA.

Amines have an atmospheric lifetime of 2–20 h^36^ and so are mainly restricted to the boundary layer. Since MSA and amines both derive from marine sources, they could be important precursors in the remote marine boundary layer or in pristine coastal areas. However, to our knowledge, nucleation of MSA with amines alone has not yet been observed in the atmosphere.

Here we present measurements of new particle formation in the CLOUD chamber at CERN involving MSA, SA, DMA and TMA under boundary layer conditions. Our experiments focus on DMA due to its higher basicity compared to methylamine and its more frequent detection in the atmosphere. We also studied TMA since it is expected to have strong marine sources and high emission rates.^23^

## 2 Methods

### 2.1 The CLOUD chamber

The results presented here were obtained during the CLOUD15 campaign at CERN (September–November 2022). The CLOUD chamber has been described in detail by previous publications, *e.g.* Kirkby *et al.*^5^ It is a 26 m^3^ stainless steel chamber equipped with various light sources and operated inside a very precisely temperature-controlled thermal housing. The chamber is characterised by its exeptionally low level of contaminants and minimal loss rates, allowing precise measurements of nucleation and growth rates to be performed at atmospheric vapour concentrations. A comprehensive array of instruments continuously samples and analyses the contents of the chamber. The experiments were performed at 60% relative humidity (RH) and at temperatures of 5 °C and −10 °C to span the typical annual range for the marine boundary layer at mid and high latitudes.

### 2.2 Trace gas and particle measurements

Gas-phase MSA, SA, DMA and TMA were measured with a Chemical Ionisation – Atmospheric Pressure interface – Time Of Flight mass spectrometer (CI-APi-TOF) using a nitrate ionisation source employing a corona discharge.^9^ The analyte molecules in the sample air are charged by deprotonation or clustering with the nitrate reagent ions (NO_3_^−^, (HNO_3_)_*n*_NO_3_^−^) and mass separated by a time-of-flight detection. Previous studies show that this measurement principle is capable of detecting SA and DMA down to very low concentrations (0.7 pptv for DMA and a 10 min averaging time,^37^ 3.6 × 10^4^ cm^−3^ for SA and a 15 min avg time^38^). In the CLOUD chamber, ions are generated by natural galactic cosmic rays (GCR). To prevent the interaction of these ions with the reagent ions and their influence on the quantification, an electric field is applied to the instrument's sampling line. Hence, only neutral molecules and clusters are measured in the CI-APi-TOF. Additionally, core sampling is used to reduce sampling losses of extremely low volatile vapours to the inlet wall.^39^

The calibration factor for the CI-APi-TOF was determined by producing a known amount of sulfuric acid from the oxidation of SO_2_ with OH, as described by Kürten *et al.*^40^. Due to the similar chemical structure of MSA and SA, the charging efficiency and hence calibration factor was assumed equal for both compounds. We expect only a minor uncertainty to result from this assumption, which is also confirmed by the calculation of the dissociation enthalpies of the clusters conducted by Shen *et al.*^12^. The estimated systematic uncertainty of the acid concentrations is a factor two, and is larger than the statistical uncertainty.

A separate calibration for DMA vapour was performed in the beginning of the campaign. The humidity-dependent calibration equation shows good agreement when comparing the measured values in the chamber with the calculated mixing ratios expected from the mass flow controller (MFC) settings and the liftetime as described by Simon *et al.*^37^ A direct calibration for TMA was not performed; hence the absolute values were calculated from the MFC settings while the instrumental data was used to confirm the qualitative trend. Details on the DMA calibration and calculation of TMA can be found in the SI (S1).

Naturally-charged ions and clusters were measured by an Aerodyne APi-TOF^41^ (positive ions) without chemical ionisation and a MION2-APi-CIMS^42^ (negative ions) by switching off the inlet voltages that direct the charged reagent ions into the sample flow.

Two scanning mobility particle sizers (SMPS)^43^ with different diameter ranges were used to determine the size distribution of the particles in the chamber for diameters between 6 and 570 nm. The nucleation rate at 1.7 nm (*J*_1.7_) was calculated using a scanning particle size magnifier (PSM)^44^ coupled to a condensation particle counter (CPC) according to Dada *et al.*^45^ The uncertainty of *J*_1.7_ results from the statistical uncertainty during each steady state and the systematic uncertainty of the instrument of 30%. The latter is typically dominant and results from the uncertainties in the formation rate (10%), the wall loss (20%), the dilution loss (10%), and the coagulation loss (20%).^45^ Additionally, a Neutral cluster and Air Ion Spectrometer (NAIS)^46^ measured small ions and charged clusters between 1 and 40 nm.

### 2.3 Experimental procedure

During each experiment the chamber was operated in a continous flow mode where the trace gases were constantly injected into the chamber to compensate for losses. To initiate an experiment, the rotation speed of the two internal mixing fans was decreased from their maximum value (100%) to 12%, which reduced the wall loss rates for ‘sticky’ vapours and molecular clusters by a factor 5. The fans are kept at this lower setting throughout the whole measurement until a cleaning is performed before starting the next experiment. In some experiments, hydroxyl radicals (OH) were generated by initiating the photolysis of ozone (O_3_) using UV illumination. After achieving a stable particle formation rate, the chamber was cleaned and prepared for the next experiment with modified conditions. The MSA concentration was varied between about 1 × 10^6^ cm^−3^ and 1 × 10^8^ cm^−3^. The sulfuric acid concentrations were lower: either below the limit-of-detection (2 × 10^4^ cm^−3^) or else between 2 × 10^5^ and 6 × 10^6^ cm^−3^. Amine mixing ratios were maintained at approximately 8 pptv for the majority of experiments, and the same mass flow controllers and injection lines were used for both DMA and TMA. DMA experiments were conducted at +5 °C and −10 °C and TMA experiments at −10 °C.

We controlled the concentrations of SA and MSA separately rather than relying on DMS oxidation. This allowed us to explore a wide range of MSA : SA ratios, which cannot be achieved through DMS oxidation alone. MSA was injected from an evaporator, and amines were obtained from pressurised bottles. These vapours were injected into the bottom of the chamber, *via* separate lines located close to the lower mixing fan. In contrast, SA was produced *in situ* by oxidation of well-mixed SO_2_ under different intensities of near-uniform UV illumination.^47^ All particle and gas sampling is done in a ‘fiducial volume’ in the mid-plane of the chamber, well away from the gas injection region.

Despite a characteristic mixing time in the chamber of around two minutes,^48^ the inhomogenity of the MSA concentration near the injection region could artificially increase the nucleation rates due to their non-linear dependence on vapour concentrations (resulting in a ‘plume’ effect). To explore a potential plume effect, a set of three experiments were conducted using fans operating at 25% of their maximum speed at high MSA concentrations (5 × 10^7^ cm^−3^), which required roughly a doubling of the MSA injection rate compared to operation at 12% fan speed. These experiments reproduced similar nucleation rates as those measured under standard mixing conditions (12% fans speed). Based on these experiments we estimate that, for the data points shown here, the uncertainty introduced by a possible plume effect is smaller than the point-to-point scatter. In the presence of SA, any plume effect for SA-MSA-amine nucleation would be strongly reduced since SA is distributed homogeneously over the entire chamber volume.

## 3 Results and discussion

### 3.1 Nucleation rates of MSA, SA and DMA

**Fig. 1:**
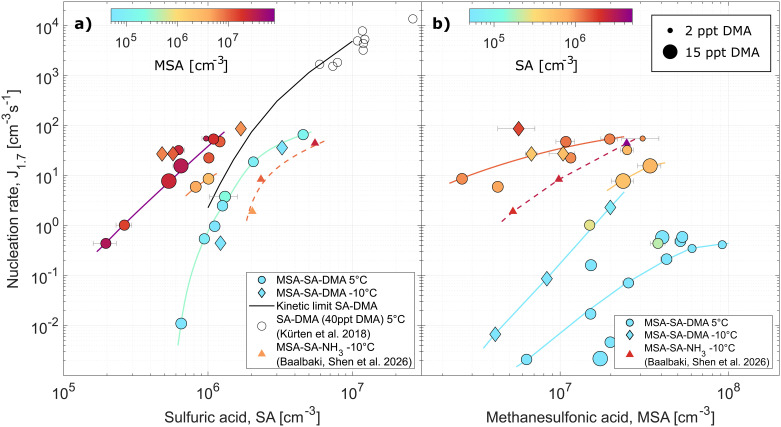
Nucleation rates *versus* acid concentrations in the presence of DMA. Nucleation rates, *J*_1.7_ as a function of (a) sulfuric acid (*x*-axis) and MSA (marker colour) concentrations and (b) MSA (*x*-axis) and sulfuric acid (marker colour) concentrations. Note different scaling in *x*-axis and colourbars in each panel. Cyan markers correspond to acid concentrations below the limit of detection and so correspond to (a) pure SA-DMA and (b) pure MSA-DMA nucleation. Circles denote the MSA-SA-DMA systems at 5 °C, diamonds denote measurements at −10 °C. MSA-SA-NH_3_ measurements at −10 °C from Baalbaki *et al.*^22^ are triangles (250–750 pptv NH_3_, acid-to-base ratio ≫1). Marker size denotes DMA mixing ratios, linearly scaled from 2 to 15 pptv. The empty triangles and the kinetic limit calculation at 5 °C are adapted from Kürten *et al.*^26^. All measurements with DMA were conducted at 60% RH at natural GCR ionisation rates. The coloured lines are drawn to guide the eye along measurements taken under similar experimental conditions. Error bars represent one standard deviation of average measurement during steady-state conditions. Systematic uncertainties, not captured in the error bars, amount to a factor 2 for acid and 30% for J rate measurements.

Fig. 1 shows our measurements of the nucleation rates, *J*_1.7_, as a function of (a) SA and (b) MSA concentrations, with the markers coloured by the different MSA and SA concentrations, respectively. The light blue markers in panel (a) without any addition of MSA are slightly lower than the kinetic limit of Kürten *et al.*,^26^ but mostly within the systematic uncertainty of the acid measurement (not shown). The addition of 3 × 10^6^ cm^−3^ MSA to 1 × 10^6^ cm^−3^ SA increases *J*_1.7_ by more than a factor 10, while higher MSA concentrations around 3 × 10^7^ cm^−3^ lead to a further enhancement of a factor 6. During experiments at lower SA concentrations, the increase in *J*_1.7_ caused by MSA was even stronger. However, the effect of MSA decreases at increasing SA concentrations.

At 5 °C, pure MSA-DMA nucleation (light blue points in Fig. 1b) yields rates below 1 cm^−3^ s^−1^, even at MSA concentrations approaching 10^8^ cm^−3^. Adding 1 × 10^6^ cm^−3^ of SA boosts nucleation rates by 2–4 orders of magnitude. This enhancement is strongest at low SA concentrations and diminishes as SA increases, where SA begins to dominate the nucleation process and MSA has only a minor effect. These results indicate that while MSA-DMA nucleation is relatively weak compared to SA-DMA at similar acid levels, MSA can significantly enhance particle formation when SA is below ∼10^6^ cm^−3^.

The DMA mixing ratio was maintained around 8 pptv for most of these experiments. Increasing DMA to 16 pptv did not significantly influence the nucleation rates. A decrease down to 2 pptv did also not lead to a substantial drop in *J*_1.7_. At 2 pptv, the DMA concentration is 5 × 10^7^ cm^−3^, hence still exceeding the acid concentration and sufficient to saturate acid–base pairing. Only when DMA becomes the limiting factor, we would expect a significant drop in the nucleation rates.

All experiments shown in Fig. 1 were conducted under GCR (galactic cosmic ray) conditions with natural ionisation rates corresponding to an ion pair production rate of 1.7 cm^−3^ s^−1^.^49^ This leads to ion concentrations representative for the boundary layer.^50^ Therefore, the influence of ions on the nucleation rate cannot be determined directly. However, measurements by the NAIS (Neutral cluster and Air Ion Spectrometer) show a clear overabundance of positive compared to negative ions for MSA-DMA nucleation (SI Fig. S2). Small positively charged clusters between 2 and 5 nm are a factor of 5–15 more abundant than their negative counterparts. This indicates that, for pure MSA-DMA nucleation, positive ion-induced nucleation dominates over negative. Quantum chemical calculations of the binding energies of the differently charged clusters would be needed to confirm an enhanced stability of the positive ions.

Upon cooling from 5 °C to −10 °C, the pure MSA-DMA nucleation rate increases by up to 50-fold, reaching 2 cm^−3^ s^−1^ at 2 × 10^7^ cm^−3^ MSA (light blue circles compared with light blue diamonds in Fig. 1b). The nucleation rates of pure SA-DMA do not change with temperature (Fig. 1a) since – in contrast with MSA-DMA – they are already proceeding at the kinetic limit.

To compare the different influence of amines and ammonia, results from Baalbaki *et al.*^22^ are included in Fig. 1. These show that significantly higher acid concentrations (approximately a factor 5 higher SA concentrations in Fig. 1) are required in the presence of ammonia than with DMA to achieve comparable nucleation rates, despite considerably higher ammonia mixing ratios. This difference is attributed to the stronger interactions between DMA and the acid, resulting from the stronger basicity of DMA compared to ammonia.

**Fig. 2:**
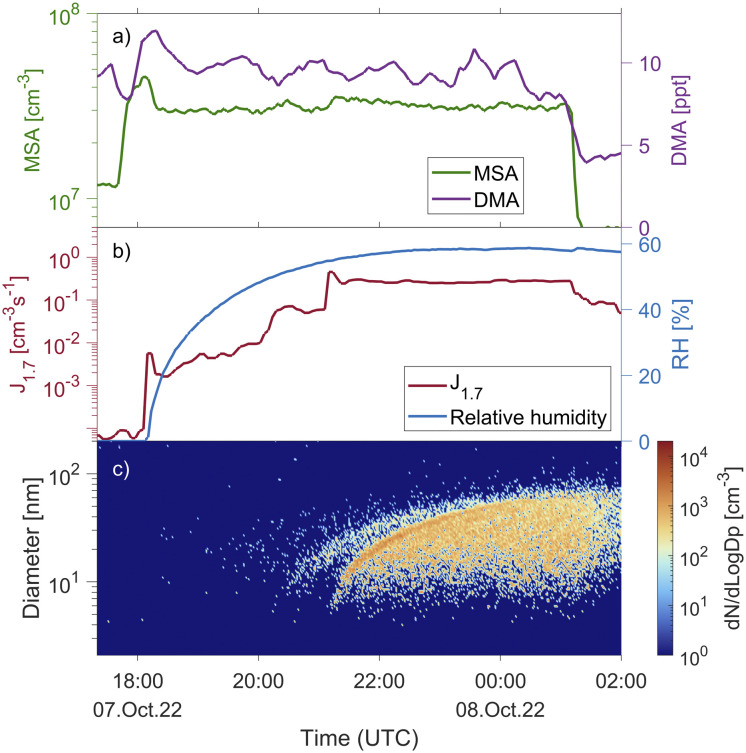
Nucleation rates during a relative humidity ramp. Evolution of an experiment at 5 °C showing (a) MSA and DMA concentrations measured by the CI-APi-TOF, (b) a ramp of the relative humidity from zero to 58%, and the corresponding nucleation rates at 1.7 nm, *J*_1.7_, and (c) particle size distribution measured by two SMPS instruments.

We observe pure MSA-DMA nucleation to have a strong dependence on relative humidity. Fig. 2 shows the nucleation rates measured during an experiment where the relative humidity was slowly increased from 0 to almost 60% at 5 °C, while MSA and DMA were kept constant at around 3 × 10^7^ cm^−3^ and 10 pptv, respectively. This change of relative humidity increases the nucleation rate by more than two orders of magnitude. Small variations in the MSA concentration are seen to cause corresponding changes in the nucleation rate. This experiment illustrates the key role of water molecules for stabilising the MSA-DMA clusters against evaporation, consistent with previous findings.^20,31^ Water molecules help stabilising the small clusters at the critical size by hydrogen bonds with MSA and DMA, as confirmed by model calculations in Dawson *et al.*^31^

### 3.2 Nucleation mechanism for MSA, SA and DMA

**Fig. 3:**
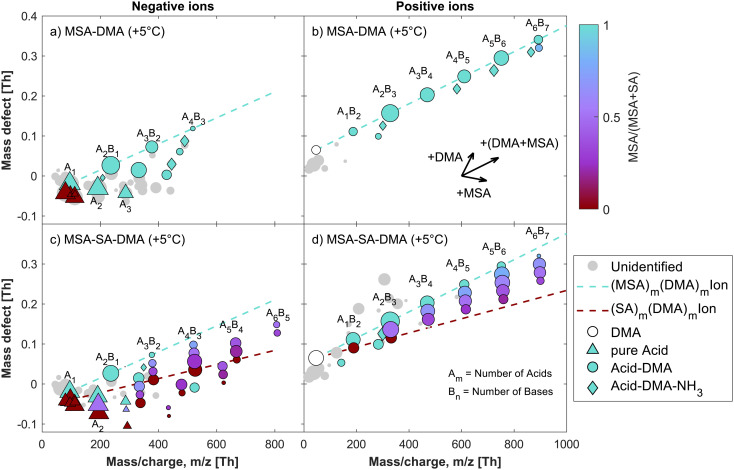
Molecular composition of charged clusters during (SA-)MSA-DMA nucleation. Mass defect (difference from integer mass) *versus m*/*z* (Th) during nucleation events at 5 °C and 60% RH for two experiments: (a) and (b) 5.2 × 10^7^ cm^−3^ MSA, 7.8 pptv DMA and a *J*_1.7_ of 0.5 cm^−3^ s^−1^; (c) and (d) 1.2 × 10^7^ cm^−3^ MSA, 1.0 × 10^6^ cm^−3^ SA, 7.5 pptv DMA and a *J*_1.7_ of 22.6 cm^−3^ s^−1^. The left panels show negative ions measured with the MION2-APi, and the right panels show positive ions measured with the APi-TOF. The negative clusters always contain a deprotonated acid and the positive ions a protonated DMA molecule. The symbol shape indicates the chemical composition and the colour shows the MSA fraction. The marker size is proportional to the logarithm of the signal rate (counts s^−1^). Dashed lines for a 1 : 1 neutral acid : base molecular ratio are drawn to guide the eye. Note that ion in the legend here stands for the deprotonated acid in (a) and (c) and the protonated base (b) and (d). The number of *m* acid and *n* base molecules in each cluster is indicated by labels A_*m*_B_*n*_, with an ion counted as its corresponding molecule. The grey markers show unidentified peaks.

Fig. 3 shows mass defect plots for charged clusters in the chamber during two nucleation events. A 45 minute averaging period was selected after the nucleation rate had stabilised and vapours had reached their steady-state concentrations. The negative charge is always associated with a CH_3_SO_3_^−^ or HSO_4_^−^ ion, whereas the positive charge is associated with a protonated DMA molecule ((CH_3_)_2_NH_2_^+^). The negatively-charged clusters are only stable if there are at least as many neutral acid molecules as bases present since the deprotonated acid ion acts as a Lewis base and prevents the attachment of excess bases. Similarly, the positive clusters are most stable if they contain the same amount of neutral acids and bases. We observed only two exceptions to this acid–base parity rule for positive clusters, each involving an equal number of acids and bases: ((MSA)_1_(DMA)_1_H+ and (MSA)_2_(DMA)_2_H+ (Fig. 3b and d). While cluster fragmentation or evaporation of molecules within the instruments' inlets is possible (as discussed in Bianchi *et al.*^51^), the compounds seen in Fig. 3 represent the most stable clusters, which drive nucleation.

Negative ion-induced nucleation of pure MSA-DMA proceeds from a negatively-charged monomer, dimer or trimer of pure MSA (Fig. 3a). The clusters then grow by addition of DMA molecules and further MSA molecules. Various combinations of acids and bases can be seen, with the strongest signal from clusters with an acid–base ratio of one. In contrast with MSA-NH_3_ nucleation^22^ pure MSA-DMA clusters can be found even at temperatures above 0 °C, indicating the higher binding strength of DMA compared with NH_3_. The pattern of cluster compositions is similar to that of SA-DMA^51^ with the exception that the MSA dimer is already found with one DMA molecule, as also observed for MSA-NH_3_.^22^

Positive ion-induced nucleation of pure MSA-DMA starts from a (CH_3_)_2_NH_2_^+^ ion and proceeds by the attachment of MSA and DMA in a strict 1 : 1 ratio (Fig. 3b). This can take place by sequential addition of monomers or by addition of MSA-DMA dimers. A small signal from contaminant ammonia is also seen in both the positive and negative clusters (diamond symbols). The ammonia concentration was measured by a Proton-Transfer-Reaction mass spectrometer (PTR). However, due to contamination in the instrument only a relatively high upper limit could be established: 180 pptv at 5 °C and 80 pptv at −10 °C. The actual ammonia mixing ratio is certainly much lower, and probably around 10 pptv or below, based on previous measurements and its weak signal in Fig. 3. Regardless of the actual concentration of contaminant ammonia, Fig. 3 shows that DMA is the dominant base driving nucleation in these events.

When sulfuric acid is added to the MSA-DMA system, the nucleation path changes dramatically to proceed *via* multi-acid MSA-SA-DMA clusters (Fig. 3c and d). In the negatively-charged channel, pure acid clusters up to the trimer are observed, containing only SA, or only MSA, or mixtures of SA and MSA. In addition to HSO_4_^−^, the SA-related ions include SO_3_^−^, SO_4_^−^, SO_5_^−^ and HSO_5_^−^; the latter, however, do not participate in nucleation. Nucleation proceeds by the attachment of SA, MSA and DMA, while maintaining an acid : base molecular ratio of 1 : 1 (Fig. 3c). The absence of a (SA)_2_(DMA)_1_ cluster (Fig. 3c) was previously observed in the pure SA-DMA system and is due to HSO_4_^−^ being a strong Lewis base, which favours pairing with a sulfuric acid molecule rather than an amine.^8^ The presence of the (MSA)_2_(DMA)_1_ cluster is therefore likely due to CH_3_SO_3_^−^ being a weaker Lewis base and hence allowing the addition of a base already to the acid dimer.

In the initial negatively-charged clusters, MSA seems to dominate over SA due to its higher gas-phase abundance, but at higher masses (above 400 Th), SA takes over and is present more strongly than MSA, as seen by the decrease in the MSA fraction. Despite the sulfuric acid concentration being more than one order of magnitude lower than the MSA concentration, the higher cluster forming potential of the stronger acid is emphasised. This is due to the higher stability of SA-DMA compared to MSA-DMA bonds.

In the positively-charged channel, MSA-SA-DMA nucleation again shows the characteristic addition of acid and base in a 1 : 1 ratio, regardless of the acid composition (Fig. 3d). The ratio of SA to MSA again increases with cluster size (see colourbar). Nevertheless, the dominant nucleation pathway clearly involves multi-acid clusters of MSA-SA-DMA. At relative MSA:SA concentrations of around 10 : 1, our measurements show that the two acids have similar molar fractions in the molecular clusters, and readily mix together with DMA to drive rapid particle nucleation.

### 3.3 Nucleation rates and mechanism for TMA

**Fig. 4:**
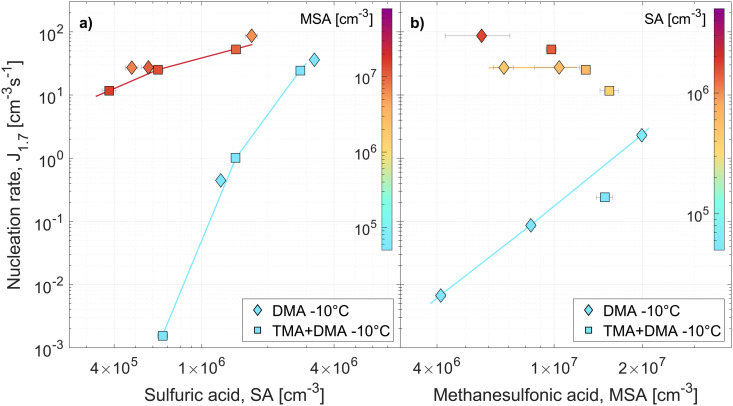
Comparison of nucleation potential from TMA and DMA in the (SA-)MSA-TMA NPF experiment at −10 °C. Nucleation rate, *J*_1.7_, *versus* (a) SA and (b) MSA concentration, with the markers coloured by their MSA and SA concentrations, respectively (note the different colour scales). The squares indicate experiments with 10 pptv trimethylamine (TMA) and 1 pptv residual DMA, while the diamonds represent pure DMA runs at a mixing ratio of 8 pptv (without any TMA addition). Lines at approximately constant acid concentrations are drawn to provide a visual reference. All measurements shown here were made with natural ionisation rates. The error bars represent the standard deviation at experimental steady-state conditions.

Atmospheric amines have a variety of molecular structures, which influence their ability to nucleate. To investigate this, we performed some experiments with MSA, SA and trimethylamine (TMA) at −10 °C. Fig. 4 shows our measured nucleation rates for 10 pptv TMA with 1 pptv residual DMA (squares), compared with our previous measurements with 8 pptv DMA (diamond symbols). Within experimental uncertainties and for this range of SA and MSA concentrations, we find no difference in nucleation rates under these two different amine conditions. However, 1 pptv (2 × 10^7^ cm^−3^) residual DMA is significant for the TMA experiments and so it is necessary also to consider the molecular composition of the clusters.

**Fig. 5:**
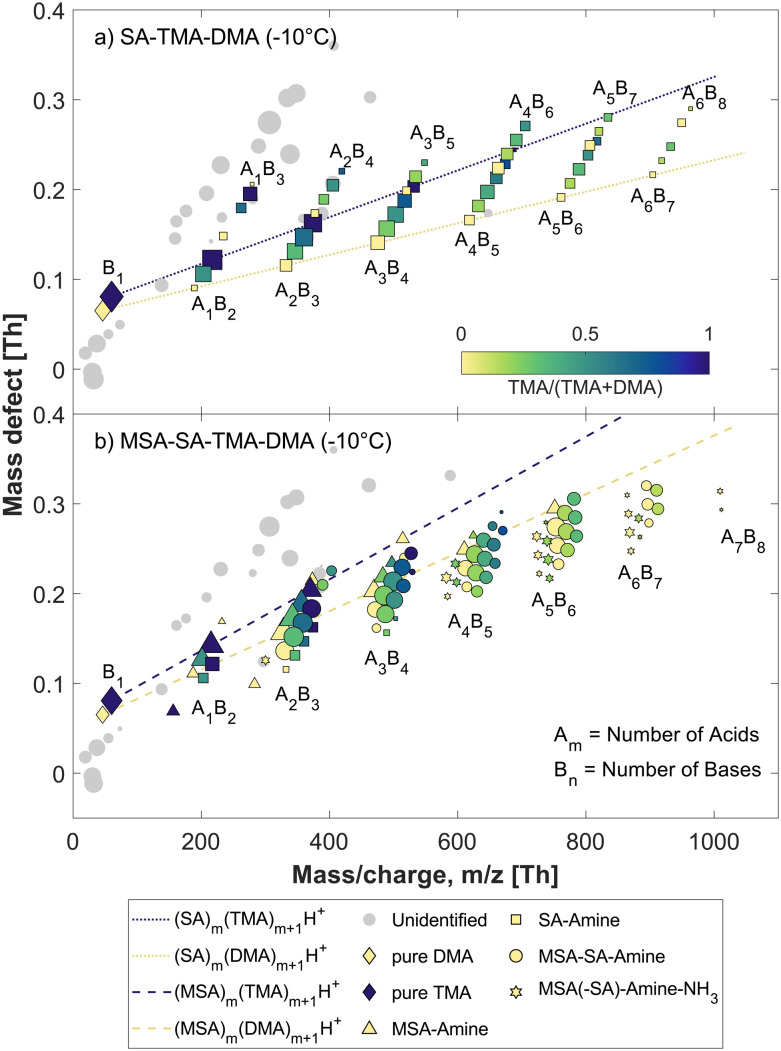
Mass defect plots of positive ions detected by the APi-TOF for two experiments with TMA at −10 °C and 60% RH. Figure (a) shows ion clusters from a mixture of 2.8 × 10^6^ cm^−3^ SA and around 10 pptv TMA with a *J*_1.7_ of 24.4 cm^−3^ s^−1^. For the experiment in (b) 1.2 × 10^7^ cm^−3^ of MSA, 6.4 × 10^5^ cm^−3^ SA and 10 pptv TMA were present. DMA was not injected directly while residual mixing ratios have upper limits of 0.9 pptv in panel (a) and 1.5 pptv in (b) respectively. The cluster compositions are indicated by the marker shape while the colour shows the TMA/DMA fraction. The size is logarithmically scaled to the peak intensity in the mass spectrum. The dashed lines show the addition of acid and base in a 1 : 1 ratio to the core ion. The number of *m* acid and *n* base molecules in each cluster is indicated by labels A_*m*_B_*n*_, with an ion counted as its corresponding molecule. Grey markers indicate unidentified peaks.

Fig. 5 shows the positive ions and particles measured during two experiments with 10 pptv TMA and acid concentrations of (a) 2.9 × 10^6^ cm^−3^ SA and (b) 1.2 × 10^7^ cm^−3^ of MSA and 6.4 × 10^5^ cm^−3^ SA. Contaminant DMA was present for these experiments, with measured upper limits of (a) 0.9 pptv and (b) 1.5 pptv, respectively. During the experiment without MSA (Fig. 5a), clusters with SA and TMA, SA and DMA as well as mixtures of SA with both amines are observed. TMA clusters dominate in the low mass range (below 400 Th) while DMA dominates at higher masses, as seen by the shift to lighter colours. There is a clear absence of pure TMA-acid clusters at high masses, whereas SA-DMA clusters are identified up to (SA)_6_(DMA)_8_. This indicates that the additional methyl group of TMA represents a significant drawback in the nucleation process. Another notable feature is the presence of clusters with two amines more than acids (seen by the second sequence shifted upwards in Fig. 5a), which is not observed in the SA-MSA-TMA system (Fig. 5b). This suggests a tendency to fully protolyse the H_2_SO_4_ molecules.

In the mixed-acid system with MSA, SA and TMA, various different clusters are detected that include all possible combinations of acids and amines (Fig. 5b). TMA dominates in the initial steps since (MSA)_1_(TMA)_2_ is the dominant positive cluster with one acid and two bases. However, there is an increasing presence of DMA as the clusters grow, despite a concentration of DMA around one tenth that of TMA. At the higher masses (above A_3_B_4_), no pure TMA clusters are observed and the spectrum is dominated by mixed amine and acid clusters. A larger signal of (contaminant) ammonia-containing clusters is detected compared with the SA-TMA experiment, similar to the pure DMA-acid case. The additional methyl group of TMA may enhance the importance of small ammonia molecules for stabilising clusters. MSA is the dominant acid in the clusters, but there remains a high fraction of sulfuric acid despite a SA : MSA gas phase ratio of only 0.05. For example, the A_1_B_2_ cluster consists of 9.6% SA, whereas the A_5_B_2_ cluster already contains about 30% SA.

In summary, although our nucleation rate measurements (Fig. 4) suggest that TMA and DMA are equivalent in promoting acid–base nucleation, the mass defect plots (Fig. 5), show that the small (1 pptv) residual DMA in these experiments was primarily responsible for driving cluster formation. The higher gas-phase basicity of TMA compared to DMA seems to be outweighed by the steric hindrance of the additional methyl group and the reduced ability to form hydrogen bonds with the acids. This is especially important in the MSA system, where also the acid has an additional methyl group. Consequently, TMA is only detected at high concentrations in small clusters (containing up to 5 monomer molecules) and contributes marginally to larger clusters with 6 or more monomers. These results are confirmed by the negative ion composition shown in the SI (S3).

## 4 Conclusions

Our study shows that MSA nucleates with DMA at temperatures of 5 °C and below, at atmospheric concentrations and without requiring any additional trace gases. Nevertheless, the nucleation rates are slow, barely reaching 1 cm^−3^ s^−1^ at MSA concentrations in the upper atmospheric range. However, at more typical atmospheric MSA levels (around 10^7^ cm^−3^) and when sulfuric acid is also present at low concentrations around 10^6^ cm^−3^, the nucleation rates reach almost 80 cm^−3^ s^−1^, far exceeding those measured for SA-DMA alone. Lower acid and base concentrations are required to reach similar nucleation rates in the presence of DMA compared to ammonia.^22^ Our molecular cluster measurements at 5 °C and −10 °C show that the dominant nucleation pathway involves multi-acid clusters containing all three molecules (SA, MSA and DMA), confirming that MSA is directly participating in the early process of nucleation and not merely contributing to particle growth at small sizes.

We further find that MSA-driven nucleation has a strong dependence on relative humidity. The dependence seems to be stronger than indicated by Chen *et al.*^32^. While structurally similar to SA, MSA contains a methyl group in place of one acidic proton, which has an inductive effect and also reduces its hydrogen-bonding capacity. This likely explains why MSA acts as a weaker nucleator and requires approximately tenfold higher concentrations to attain molar fractions comparable to those of SA in molecular clusters.

Our measurements of the composition of charged clusters in the presence of 10 pptv TMA and around 1 pptv DMA show that TMA is less efficient than DMA for acid–base nucleation involving either MSA or SA. Although TMA is the stronger base in the gas phase, this does not compensate for its steric hindrance and reduced ability to form hydrogen bonds due to an extra methyl group, resulting in less solvation than its counterpart DMA and making it the weaker base in the condensed phase. We expect that in the absence of other bases TMA will show lower nucleation rates than DMA, likely not reaching the kinetic limit with SA. As such, higher TMA concentrations will be required to drive cluster formation and nucleation compared to DMA or methylamine.

From a simplified perspective, the nucleation mechanism is governed by three main features: (1) the binding strength determined by the gas-phase acidity/basicity which is higher for SA and TMA compared to MSA and DMA, (2) the hydrogen bonding capacity which favours SA and DMA and (3) the concentration of the vapours which depends on the atmospheric conditions but could favour MSA in remote marine areas. It appears that the lower hydrogen bonding capacities of MSA and TMA do not represent a drawback in the initial steps of dimer or trimer formation but become more and more obstructive as the clusters grow towards the critical size.

The synergistic effect of MSA and SA, along with the lower nucleation potential of MSA, are in good agreement with other publications studying these compounds in flow tubes^20,21,32,52^ or model simulations.^35^ We confirm with our measurements that these effects lead to significant particle formation rates under atmospheric concentrations and conditions of the marine boundary layer in mid latitudes. While the temperature dependence of the nucleation rate has been studied before^20^ we provide the first measurements conducted significantly below room temperature. Additionally, this is the first study to report measurements of clusters with up to 15 monomers. Their chemical composition agrees well with theoretical studies.^35,53^ While TMA has initially been reported to be similarly effective in nucleation as DMA in combination with MSA^32,35^ or SA,^21,33^ our results suggest a decreased nucleation potential aligning with newer or updated studies.^53,54^ A summary of previous studies and their experimental conditions can be found in the SI (S4).

The oxidation of dimethyl sulfide (DMS) from phytoplankton produces both MSA and SA, so they are generally found together in marine environments. Our study shows that these two acids act synergistically to drive rapid new particle formation, in the presence of minute levels of DMA, which have been found by multiple studies in the marine boundary layer. Pristine marine regions are especially sensitive to cloud condensation nuclei from secondary particle formation due to the generally lower availability of CCN.^55^ The MSA-SA-amine nucleation investigated here under clean chamber conditions may apply directly to marine atmospheres when the condensation sink is low, such as after precipitation events. When background aerosols are present (from sea spray or advective transport), this high condensation sink can shift the system from a nucleating case to one contributing to growth of existing aerosols. The marine environment also contains a rich mixture of other condensable vapours (iodic acid, organics) which may contribute to nucleation and growth with strongly synergistic effects, lowering the concentrations required for each of the compounds to initiate nucleation.

Capturing aerosol formation and growth in these critical regions demands expansion into this multi-component parameter space in future studies. For the MSA-amine system also further investigations across a range of temperatures and relative humidities are needed. These are essential to derive a robust parametrisation suitable for implementation in atmospheric models. Future climate models will need to account for MSA-driven nucleation both to understand current aerosol radiative forcing and to reliably project future warming as anthropogenic aerosol may decrease in response to air quality policies giving natural aerosol sources more importance again.

## Author contributions

H. K., L. C.-P., M. H. and J. C. planned the experiments. H. K., L. C.-P., M. H., D. M. R., B. R., R. B., J. S., E. S., D. A., J. A., A. A., H. B., M. B., T. C., L. D., J. D., N. D., J. D., I. E., X. H., H. H., F. K., M. K., K. L., B. M., T. P., S. R., P. R., W. S., S. S., M. S., J. T., Y. T., A. T., P. M. W., B. Y., M. Z-W., J. Z., J. K., and J. C. prepared the CLOUD facility and measurement instruments. H. K., L. C.-P., M. H., D. M. R., B. R., R. B., J. S., E. S., J. A., A. A., D. A., H. B., M. B., L. D., J. D., I. E., X. H., H. H., B. M., P. R., S. R., Y. T., J. T., A. T., R. C. T., B. Y., M. Z.-W., and J. K. collected the data. H. K., L. C.-P., B. R., E. S., A. K., X. H., L. L., and B. Y. analyzed the data. H. K., L. C-P., M. H., D. M. R., J. S., E. S., A. K., J. A., L. J. B., N. D., J. D., I. E., A. H., X. H., M. K., K. L., M. S., R. V., J. K., and J. C. contributed to the scientific discussion. H. K., D. M. R., J. K., and J. C. contributed to writing the manuscript.

## Conflicts of interest

There are no conflicts to declare.

## Supplementary Material

EA-006-D5EA00081E-s001

## Data Availability

Data for this article, including all data shown in the figures in the main text and SI, are available on Zenodo at https://doi.org/10.5281/zenodo.15861508. Supplementary information is available. See DOI: https://doi.org/10.1039/d5ea00081e.
