# Supplementary material for: Rapid new particle formation driven by methanesulfonic acid and amines
Source: Environ Sci Atmos. 2026 Jul 6;6(7):1028–39. doi: 10.1039/d5ea00081e (PMC13334493; doi:10.1039/d5ea00081e)
Supplement: EA-006-D5EA00081E-s001 [file EA-006-D5EA00081E-s001.pdf]

Supplementary Information (SI) for Environmental Science: Atmospheres.
This journal is © The Royal Society of Chemistry 2025

Supporting Information for:

Rapid new particle formation driven by methanesulfonic acid and amines

Hannah Klebach[a], Lucía Caudillo-Plath[a], Martin Heinritzi[a], Douglas M. Russell[a], Birte Rörup[b], Rima Baalbaki[b,c], Jiali Shen[b,d], Eva Sommer[e,f], Andreas Kürten[a], Dina Alfaouri[b], Joao Almeida[e,g], António Amorim[g,h], Lisa J. Beck[a], Hannah Beckmann[i], Moritz Berntheusel[a], Theodoros Christoudias[c], Lubna Dada[j], Jenna DeVivo[k], Neil Donahue[k], Jonathan Duplissy[b], Imad El Haddad[j], Armin Hansel[i], Hartwig Harder[l], Xu-Cheng He[b], Markku Kulmala[b], Felix Kunkler[l], Katrianne Lehtipalo[b,m], Lu Liu[j], Bernhard Mentler[n], Tuukka Petäjä[b], Pedro Rato[a,e], Sarah Richter[a], Siegfried Schobesberger[o], Wiebke Scholz[n], Mario Simon[a], Roseline C. Thakur[b], António Tomé[o], Yandong Tong[p], Jens Top[j], Rainer Volkamer[p], Paul M. Winkler[f], Boxing Yang[j], Marcel Zauner-Wieczorek[a], Jiangyi Zhang[b], Jasper Kirkby[a,e] and Joachim Curtius[a]

[a] Institute for Atmospheric and Environmental Sciences, Goethe University Frankfurt, Frankfurt am Main, Germany
[b] Institute for Atmospheric and Earth System Research/Physics, Faculty of Science, University of Helsinki, Helsinki, Finland
[c] Climate and Atmosphere Research Centre (CARE-C), The Cyprus Institute, Nicosia, Cyprus
[d] Helsinki Institute of Physics, University of Helsinki, Helsinki, Finland.
[e] CERN, the European Organization for Nuclear Research, Geneva, Switzerland
[f] Faculty of Physics, University of Vienna, Wien, Austria
[g] Faculdade de Ciências da Universidade de Lisboa, Lisboa, Portugal
[h] Laboratório de Instrumentação e física experimental de Partículas, Lisboa, Portugal
[i] Institute for Ion Physics and Applied Physics, University of Innsbruck, Innsbruck, Austria
[j] Laboratory of Atmospheric Chemistry, Paul Scherrer Institute, Villigen, Switzerland
[k] Center for Atmospheric Particle Studies, Carnegie Mellon University, Pittsburgh, PA, USA
[l] Atmospheric Chemistry Department, Max Planck Institute for Chemistry, Mainz, Germany
[m] Finnish Meteorological Institute, Helsinki, Finland
[n] Department of Technical Physics, University of Eastern Finland, Kuopio, Finland
[o] Instituto Dom Luiz (IDL), Universidade da Beira Interior, Covilhã, Portugal
[p] Department of Chemistry, University of Colorado Boulder, Boulder, CO, USA

S1. Detection of amines by the nitrate CI-APi-TOF

DMA

DMA is detected by the nitrate CI-APi-TOF mainly clustered with the reagent ion dimer ($(CH_3)_2NHHNO_3NO_3^-$) at m/z 170 Th. A smaller signal for the cluster with the reagent ion trimer could also be detected at m/z 233 Th but was excluded in the analysis due to the high noise level. A calibration for DMA was conducted before the campaign using DMA from a gas bottle and a two step dilution system with a humidifier. Two different concentrations and three different humidities were used to determine the calibration equation in (1).

$$C_{DMA} = \frac{1153.9\,\mathrm{ncps\,ppt}}{2.16\times10^{-20}\,\mathrm{ncps\,cm^3}\cdot[\mathrm{H_2O}] + 1.06\times10^{-4}\,\mathrm{ncps}} \quad (1)$$

$$[\mathrm{H_2O}] = \frac{N_A\,p(T)}{R\,T} = \frac{N_A\,p_s(T_d)}{R\,T} = \frac{N_A}{R\,T[\mathrm{K}]}\,611.2\,\exp\frac{18.678\,T_d[^\circ\mathrm{C}]}{257.14 + T_d[^\circ\mathrm{C}]} \quad (2)$$

The DMA mixing ratio in the chamber can also be calculated from the MFC settings, the gas bottle concentration VMR_B, the volume of the chamber V_{ch}, the wall and dilution loss terms as shown by Simon *et al.* [1] using equation 3. The wall loss of DMA was determined experimentally and yielded a value of $3.7\times10^{-3}\,\mathrm{s^{-1}}$ at 5°C. The comparison of calculated and measured mixing ratios shows good agreement even during periods with highly variable humidity as shown in Fig. S1. The detection limits at the two temperatures using different averaging times can be found in

table S1.

$$\mathrm{VMR_{DMA}} = \frac{\mathrm{MFC1} \cdot \mathrm{MFC3}}{\mathrm{MFC1} + \mathrm{MFC2}} \cdot \frac{\mathrm{VMR}_B \cdot 10^{12}\,\mathrm{ppt}}{V_{ch} \cdot (k_{wall} + k_{dil})} \tag{3}$$

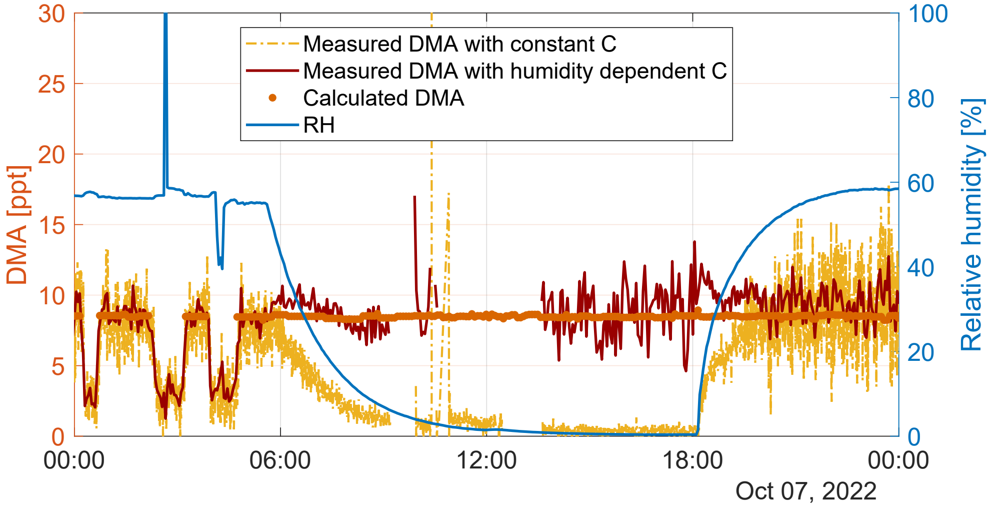

Figure S1. Measured and calculated DMA mixing ratios in the CLOUD chamber during a decrease and increase of the relative humidity. The data gap between 09:00 and 14:00 is due to instrumental problems. The yellow line shows the DMA mixing ratio if a constant calibration factor is assumed while the red line shows the results using the determined humidity dependent factor. The injection of DMA was kept constant as shown by the calculated values.

Temperature	Averaging time	Limit of detection
5 °C	30 s	2.22 ppt
	3 min	0.96 ppt
−10 °C	30 s	1.18 ppt
	3 min	0.51 ppt

Table S1. Limit of detection for DMA

TMA

For TMA no direct calibration was possible but similar to DMA the mixing ratio could be calculated from the MFC settings and the loss terms. The wall can be assumed to be a perfect sink for TMA due to the high abundance of acids on the wall and the cold temperature (-10°C). Therefore, the wall loss rate of DMA can be used to estimate the wall loss of TMA based on the diffusion coefficients of both compounds [1, 2] and equation 5 [3, 4].

$$k_{wall,\mathrm{TMA},-10^\circ\mathrm{C}} = k_{wall,\mathrm{DMA},5^\circ\mathrm{C}} \left(\frac{263K}{278K}\right)^{0.875} \sqrt{\frac{D_{\mathrm{TMA}}}{D_{\mathrm{DMA}}}} \tag{4}$$

(5)

S2. Charge imbalance in clusters

Analysis of the APi-TOF and MION2-APi spectra revealed significantly more clusters for the MSA-DMA system up to higher masses in positive than in negative mode. If sulfuric acid was present this could also be observed but to a lesser extent. To confirm this observation the data of the Neutral cluster and Air Ion Spectrometer (NAIS) can be used [5]. The data for an experiment with $5.1 \times 10^7\,\mathrm{cm^{-3}}$ MSA, 7.8 ppt DMA and a $J_{1.7}$ of $0.5\,\mathrm{cm^{-3}\,s^{-1}}$can be seen in Fig. S2, where a) shows the positive ion size distribution b) the negative ion size distribution and c) concentration of ions in two different size ranges. Note that the small ion band present below 2 nm is not shown in a) and b) to restrict the colourbar range. It can be clearly seen that the positive ions have a much higher concentration at the

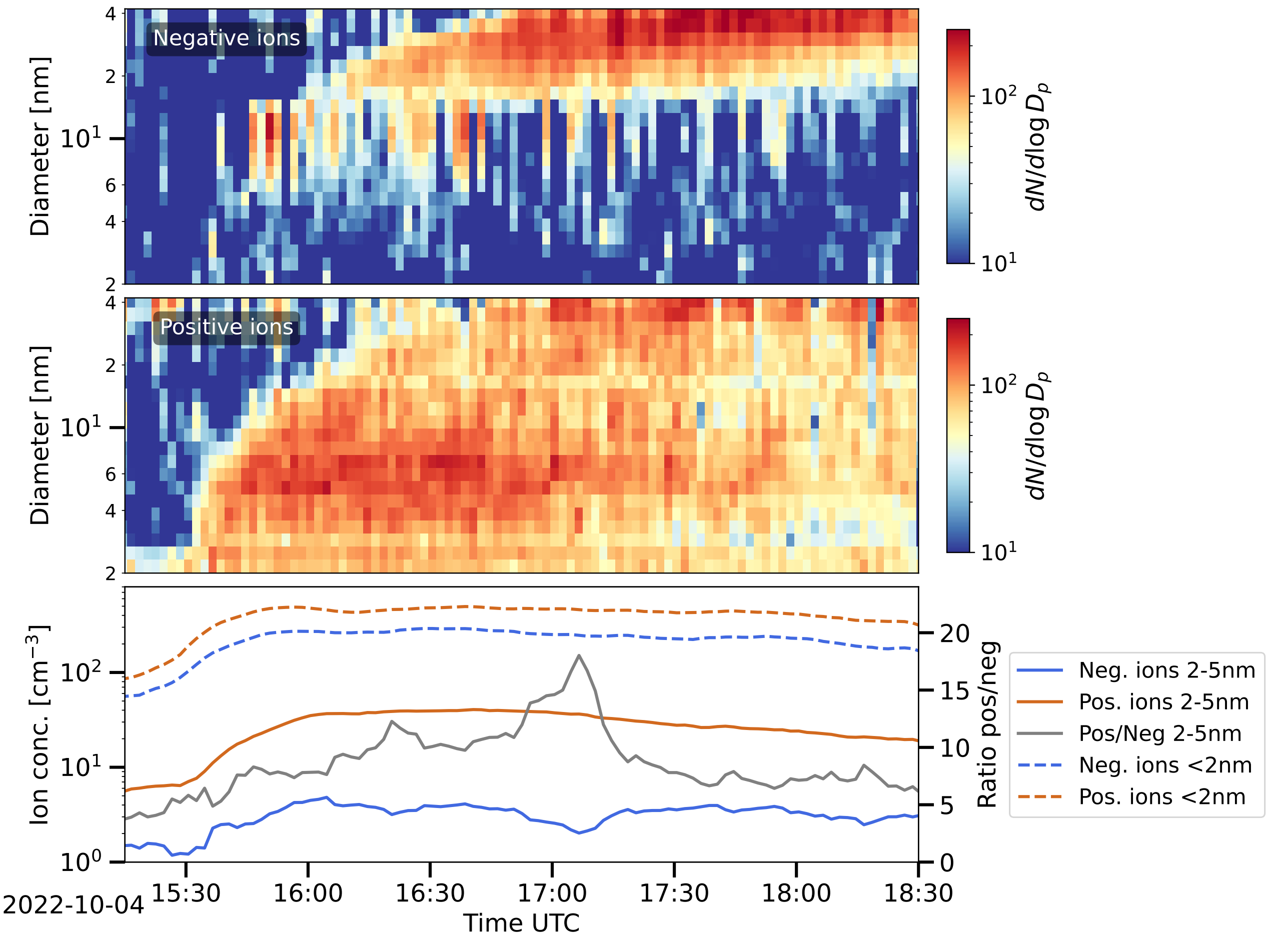

Figure S2. NAIS data for the positive and negative ions during a nucleation experiment with $5.1 \times 10^7\,\mathrm{cm}^{-3}$ MSA and 7.8 ppt DMA. The first and second panel show size distributions of the negative and positive ions and small clusters. The third panel shows the moving average of the absolute concentration of the ions in two size ranges: below 2 nm and 2-5 nm as well as the ratio for the larger size range.

initial small clusters and show a growth towards larger sizes as expected in a nucleation event. For the negative ions on the other hand very low concentrations are observed in the small sizes, higher values can only be observed later and above a diameter of 20 nm. This indicates that the nucleation is predominantly driven by positive ions and the negative ions appear mainly as a result of diffusion charging. The exact reason for this observation is not fully understood, it seems however that positively charged clusters are more stable than negatively charged ones. It could also be related to the fact that DMA is in overabundance compared to MSA and hence a collision with a positive DMA ion is more likely. The important difference to the SA-DMA system is that the nucleation does not proceed at the kinetic limit and the clusters are less stable which would indicate a stronger influence of ions as stabilizing agents. Experiments under different ion conditions are needed to varify this hypothesis.

S3. Negative clusters in the TMA system

Figure S3 shows the negative clusters recorded by the MION2-APi instrument during experiments with SA, TMA and residual DMA (a), as well as MSA, SA, TMA, and residual DMA (b). The same experiments as in Figure 5 of the main text are shown.

For the SA-DMA-TMA nucleation, mainly clusters with SA and DMA are observed. All clusters contain at least

as many neutral SA molecules as bases. In addition to HSO_4^-, we also observe a rather strong signal of clusters charged by HSO_5^-, as indicated by the red dots which was already discussed in previous studies [6, 7]. This was also observed at -10°C with DMA (not shown) and hence could be a result of the lower temperature. The nucleation mechanism proceeds in the same way regardless of the charging ion. The contribution of TMA is very small despite the tenfold higher mixing ratio compared to DMA. This is similar to, but even more pronounced than, what is seen in the positive clusters (Fig. 5, main text), which strongly emphasises the much higher nucleation potential of DMA.

The system of MSA, SA, TMA and DMA introduces a significant complication in the analysis of the mass spectra. The combination of $(H_2SO_4)((CH_3)_3N)HSO_4^-$ (i.e. $(SA)(TMA)HSO_4^-$) has exactly the same mass as $(CH_4SO_3)((CH_3)_2NH)HSO_5^-$ (i.e. $(MSA)(DMA)HSO_5^-$); hence both clusters cannot be distinguished by the mass spectrometer. Due to the lack of TMA in the SA experiment (a), it can be assumed that the contribution of TMA is minimal also in the experiment with MSA. Colours and marker shapes in Fig. S3 b) were determined assuming that all ambiguous clusters are composed of $(MSA)(DMA)HSO_5^-$. These should therefore be treated with caution, since the contribution of $(SA)(TMA)HSO_4^-$ cannot be quantified. Nevertheless, also here the dominance of DMA over TMA can be observed, as well as the synergistic incorporation of MSA and SA molecules.

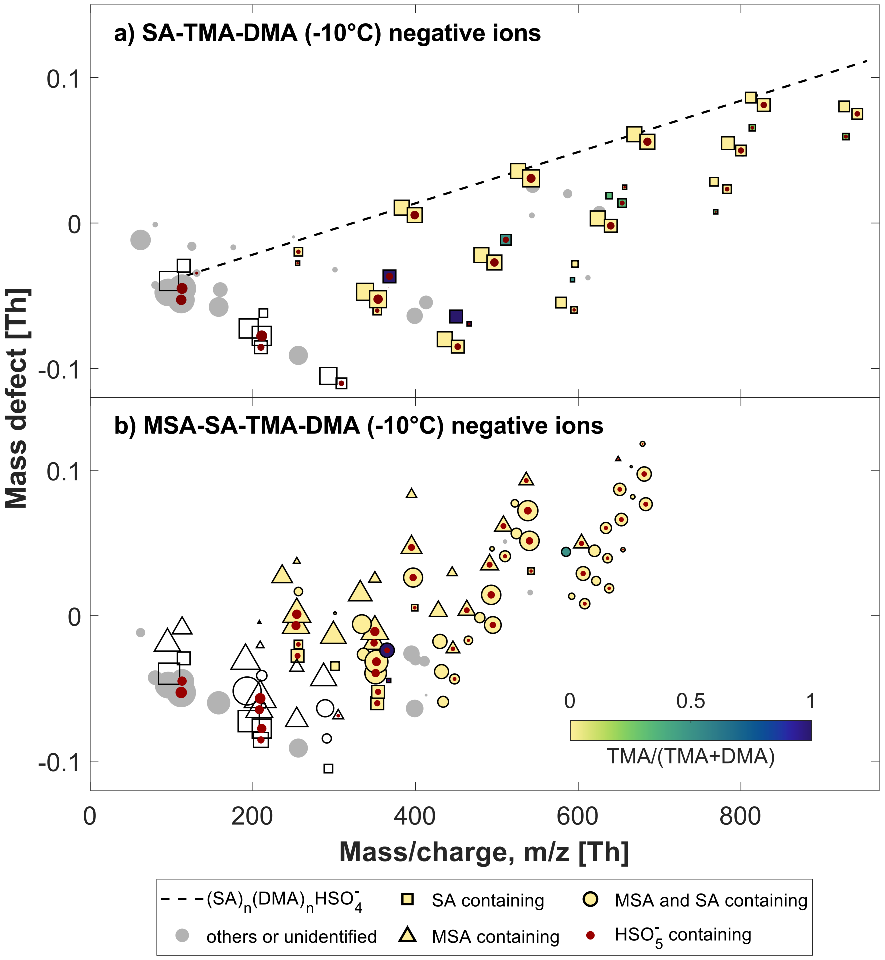

Figure S3. Mass defect plots of negative ions detected by the MION2-APi for two experiments with TMA at -10°C and 60% RH. Figure a) shows ion clusters from a mixture of $2.8 \times 10^6, cm^{-3}$ SA and around 10 ppt TMA. For the experiment in b), $1.2 \times 10^7, cm^{-3}$ MSA, $6.4 \times 10^5, cm^{-3}$ SA, and 10 ppt TMA were present. DMA was not injected directly, while residual mixing ratios were at upper limits of 0.9 ppt in panel a) and 1.5 ppt in b), respectively. The cluster compositions are indicated by the marker shape, while the colour shows the TMA fraction. The size is logarithmically scaled to the peak intensity in the mass spectrum. The dashed line in a) shows the addition of acid and base in a 1:1 ratio to the HSO_4^- ion. The clusters containing an HSO_5^- core ion are indicated by a red dot. Note that the cluster composition in b) is ambiguous, since $(H_2SO_4)((CH_3)_3N)HSO_4^-$ and $(CH_4SO_3)((CH_3)_2NH)HSO_5^-$ have the same mass and cannot be distinguished. Hence, these clusters potentially contain more TMA and SA than indicated here.

S4. Literature review

Multiple previous studies have investigated new particle formation involving MSA and amines. An overview of selected publications in the past 13 years is shown in table table S2. The following discussion provides a comparison between these publications and the current work in order to put the present study into perspective.

The publications by Dawson *et al.* [8], Chen *et al.* [9, 10], Chen and Finlayson-Pitts [11] use flow tube studies and modelling to investigate nucleation by MSA and amines. Particle formation involving only these compounds and water vapour could be confirmed. Additionally, the formation rates increase with increasing relative humidity and decreasing temperature. These findings align well with the results of this study. However, the concentrations used in the older studies are much higher than expected in the atmosphere, leading to very high J rates of more than 10000 $cm^{-3}s^{-1}$. Due to the strong non-linearity, a direct interpolation to lower concentrations is not feasible.

The studies were all conducted approximately at room temperature. Chen and Finlayson-Pitts [11] explored different temperatures, though these lie in a rather narrow range of 21-28°C. With values of +5°C and -10°C the present study focuses on temperatures of the marine boundary layer of mid and high latitudes, where MSA is expected to most strongly impact particle formation. Unlike the first three studies in table S2, our study also involves SA which will almost certainly be present in the atmosphere simultaneously with MSA due to the same chemical source DMS. We show that for high SA concentrations this acid will dominate the particle formation, and the effect of temperature will be reduced.

There are also some significant differences in the quantitative findings. While Chen *et al.* [10] report an increase in $J_{2.5}$ of approximately a factor 2 when changing from dry to humid conditions, we observe a much stronger influence with $J_{1.7}$ increasing approximately two orders of magnitude for a change in RH from 0% to 60%. Both Chen *et al.* [10] and Chen and Finlayson-Pitts [11] initially state a similar nucleation potential for DMA and TMA, which stands in contrast to the strongly reduced TMA contribution that we see in the experiments with TMA. In fact, the nucleation seems to be driven by the presence of DMA despite the low availability. However, a correction [12] attributes the high formation rates for TMA to contaminant ammonia.

The papers by Jonas Elm [13, 14, 15] present a thorough analysis of quantum chemical calculations involving MSA, SA and different bases. This allows a good insight into the binding energies and formation of clusters for the different chemical systems. However, the formation rates reported are only potential J rates not directly applicable to the atmosphere. Additionally, the modelling was performed at room temperature and water vapour is not considered. The study shows the synergistic effect of MSA and SA at atmospheric concentrations and also recognizes the steric hindrance of TMA in combination with MSA. The results are in general in agreement with our findings. Though no steric hindrance is predicted for by Elm [13] for SA and TMA, which could result from the fact that only clusters up to 4 monomers are considered in the calculations, whereas the charged clusters in our experiments indicate that the steric hindrance becomes progressively more obstructive as the clusters grow. Our results indicate that for a correct representation of the cluster formation potential more than the initial tetramers are required. In fact, a later publication by Wu *et al.* [16] shows that while in the dimer SA-TMA is similarly strongly bound as SA-DMA the binding strength deviates strongly as more monomers are added. SA-TMA clusters are subsequently less stable and more likely to evaporate. This is consistent with our findings.

Johnson and Jen [17] provide flow tube studies as well as the measurement of acid dimers and particle concentrations to infer the particle formation efficiency, though no formation rates are reported. The acid concentrations are lower than in the other flow tube experiments but remain on the higher end of the atmospheric range. Unlike our study this one is mainly operating in a base limited system. The presence of MSA in the smallest particles, synergistic effect of MSA and SA and the steric hindrance observed for MSA-TMA are in good agreement with our results. One main difference is that Johnson and Jen [17] report strong particle formation of TMA and SA, which is suppressed by high MSA concentrations. While we observe similar J rates in the DMA and TMA experiments, we can attribute these to the presence of residual DMA. The chemical composition of the charged clusters indicates that TMA is far less efficient than DMA even in combination with SA.

In conclusion, there is good agreement with previous studies on the potential of MSA and amines to form particles, though at rates much lower than in the presence of sulfuric acid. Also the influence of humidity and temperature aligns with previous findings. The present study adds to the understanding of the system by combining atmospherically relevant precursor gas concentrations and conditions. Additionally, the measurement of charged clusters provides new insights into the nucleation mechanism and helps bridge the gap to theoretical modelling.

Table S2. Summary of selected previous publications on MSA and amine new particle formation

Publication	Method	MSA [cm^{-3}]	SA [cm^{-3}]	Base	T [°C]	RH [%]	J rates
Dawson *et al.* [8]	Flow tube, modelling	5e10–8e11	-	DMA (0–8ppb)<br>TMA (0–8ppb)	Room temp.	0–20	not reported
Chen *et al.* [9]	Flow tube, modelling	7e9-4e11	-	TMA (0.5-26ppb)	Room temp.	<2–59	$J_{2.5}$ [a]
Chen *et al.* [10]	Flow tube, modelling	5e10	-	DMA (2.5ppb)<br>TMA (2.5ppb)<br>NH3<br>Methylamine	Room temp.	<2–61	$J_{2.5}$
Chen and Finlayson-Pitts [11]	Flow tube, modelling	7e9–25e10	-	DMA (1.6–13ppb)<br>TMA (3.7–13ppb)<br>NH3	21–28	Dry and 41–42	$J_{1.4}$ or $J_{2.5}$
Elm [13]	Modelling	1e5–1e7	1e6	DMA (1–10ppt)<br>TMA (1–10ppt)<br>NH3, MA, EDA	25	Dry	potential J rates
Johnson and Jen [17]	Flow tube	1e7–1e10	1e7–1e9	DMA (∼15ppt)<br>TMA (∼15ppt)<br>NH3, MA	25–27	20	not reported
This study	Chamber	1e6–1e8	2e5–6e6	DMA (∼10ppt)<br>TMA (∼10ppt)	5 and –10	mostly 60; ramp 0–60	$J_{1.7}$

Note: [a]The high nucleation rates were attributed to NH_3 contamination in a correction Chen *et al.* [12].

References

[1] M. Simon, M. Heinritzi, S. Herzog, M. Leiminger, F. Bianchi, A. Praplan, J. Dommen, J. Curtius and A. Kürten, *Atmospheric Measurement Techniques*, 2016, **9**, 2135–2145.

[2] S. C. Mitchell and R. L. Smith, *Chemical Senses*, 2016, **41**, 275–279.

[3] J. G. Crump and J. H. Seinfeld, *Journal of Aerosol Science*, 1981, **12**, 405–415.

[4] E. N. Fuller, P. D. Schettler and J. C. Giddings, *Industrial & Engineering Chemistry*, 1966, **58**, 18–27.

[5] S. Mirme and A. Mirme, *Atmospheric Measurement Techniques*, 2013, **6**, 1061–1071.

[6] S. Schobesberger, A. Franchin, F. Bianchi, L. Rondo, J. Duplissy, A. Kürten, I. K. Ortega, A. Metzger, R. Schnitzhofer, J. Almeida, A. Amorim, J. Dommen, E. M. Dunne, M. Ehn, S. Gagné, L. Ickes, H. Junninen, A. Hansel, V.-M. Kerminen, J. Kirkby, A. Kupc, A. Laaksonen, K. Lehtipalo, S. Mathot, A. Onnela, T. Petäjä, F. Riccobono, F. D. Santos, M. Sipilä, A. Tomé, G. Tsagkogeorgas, Y. Viisanen, P. E. Wagner, D. Wimmer, J. Curtius, N. M. Donahue, U. Baltensperger, M. Kulmala and D. R. Worsnop, *Atmospheric Chemistry and Physics*, 2015, **15**, 55–78.

[7] N. T. Tsona, L. Liu, X. Zhang and L. Du, *Atmospheric Environment*, 2021, **253**, 118362.

[8] M. L. Dawson, M. E. Varner, V. Perraud, M. J. Ezell, R. B. Gerber and B. J. Finlayson-Pitts, *Proceedings of the National Academy of Sciences of the United States of America*, 2012, **109**, 18719–18724.

[9] H. Chen, M. J. Ezell, K. D. Arquero, M. E. Varner, M. L. Dawson, R. B. Gerber and B. J. Finlayson-Pitts, *Physical chemistry chemical physics : PCCP*, 2015, **17**, 13699–13709.

[10] H. Chen, M. E. Varner, R. B. Gerber and B. J. Finlayson-Pitts, *The Journal of Physical Chemistry B*, 2016, **120**, 1526–1536.

[11] H. Chen and B. J. Finlayson-Pitts, *Environmental Science & Technology*, 2017, **51**, 243–252.

[12] H. Chen, M. J. Ezell, K. D. Arquero, M. E. Varner, M. L. Dawson, R. B. Gerber and B. J. Finlayson-Pitts, *Physical chemistry chemical physics : PCCP*, 2017, **19**, 4893.

[13] J. Elm, *ACS omega*, 2021, **6**, 7804–7814.

[14] J. Elm, *ACS omega*, 2021, **6**, 17035–17044.

[15] J. Elm, *ACS omega*, 2022, **7**, 15206–15214.

[16] H. Wu, Y. Knattrup, A. B. Jensen and J. Elm, *Aerosol Research*, 2024, **2**, 303–314.

[17] J. S. Johnson and C. N. Jen, *ACS earth & space chemistry*, 2023, **7**, 653–660.
